# A low-field Nuclear Magnetic Resonance dataset of whole milk during coagulation and syneresis

**DOI:** 10.1016/j.dib.2019.104520

**Published:** 2019-09-16

**Authors:** E. Curti, A. Pardu, S. Del Vigo, R. Sanna, R. Anedda

**Affiliations:** Porto Conte Ricerche s.r.l., S.P. 55 Porto Conte-Capo Caccia, Km 8.400 Loc, Tramariglio, Alghero (SS), Italy

**Keywords:** Dairy science and technology, Milk thermization and pasteurization, Low-field NMR, T_2_ relaxation, Cheese production, Curd syneresis, Cow's and goat's milk

## Abstract

We report the relaxometric dataset obtained on renneted milk during syneresis by Time-Domain Nuclear Magnetic Resonance spectroscopy (TD-NMR). Data were obtained on cow's milk provided by two different producers in two different lactation seasons (April and October) and on a group of goat's milk samples (one season, November–December, one producer). TD-NMR data refer to spin-spin relaxation times (T_2_) decay curves and distributions measured at 40 °C at seven time points after rennet addition, up to 70 minutes of syneresis. Curd was cut 30 min after rennet addition without removing the NMR tube from the TD-NMR instrument. The dataset here reported is related to the research article entitled “Non invasive monitoring of curd syneresis upon renneting of raw and heat-treated cow's and goat's milk” [E. Curti, A. Pardu, S. Del Vigo, R. Sanna, R. Anedda, Non-invasive monitoring of curd syneresis upon renneting of raw and heat-treated cow's and goat's milk, Int. Dairy J. 90 (2019) 95–97].

**Table:** Specifications Table

Subject area	*Food Chemistry*
More specific subject area	*Dairy Science*
Type of data	*xlsx file (XY data table)*
How data was acquired	*Low-field Nuclear Magnetic Resonance Spectrometer (20* MHz*, 0.47 T, Bruker the miniSpec, Germany)*
Data format	*Raw and processed (CONTIN software, Bruker, Germany) T*_*2*_*CPMG decays*
Experimental factors	*Fresh whole milk was analyzed raw and heat-treated*
Experimental features	*1. Heat treatment of milk (72°C, 1 minute)* *2. Addition of rennet, coagulation and syneresis* *3. Acquisitions of T*_*2*_*[transverse relaxation time, Carr-Purcell-Meiboom-Gill (CPMG) pulse sequence] decays at selected syneresis time points, up to 70 minutes* *4. Analysis of T*_*2*_*data decays with CONTIN software to obtain T*_*2*_*distributions* *5. Comparison of T*_*2*_*distributions of raw and heat-treated milk and curd*
Data source location	*Porto Conte Ricerche S.r.l., Tramariglio, Alghero (SS), Italy*
Data accessibility	*Data are included in this article, as a supplementary file*
Related research article	*Curti, E. Pardu, A., Del Vigo, S. Sanna, R., and Anedda R. Non invasive monitoring of curd syneresis upon renneting of raw and heat-treated cow's and goat's milk. Int. Dairy J.* 90 (2019) 95–97. https://doi.org/10.1016/j.idairyj.2018.11.003

**Table:** 

**Value of the Data** • The dataset is useful to demonstrate and understand the effect of heat treatments of milk on the microstructural features of curd during syneresis • The dataset can be useful to both researchers involved in the application of NMR relaxometry in dairy science and to dairy technologists • The dataset can be used as a comparison in studies investigating the effect of other parameters (e.g. season, cattle diet, cattle species, rearing environment, climate) on milk and curd microstructural features. • To the best of our knowledge, this is the first published NMR relaxometry dataset on whole milk • Our dataset would serve as a starting point for the implementation of NMR in process and quality control of dairy products

## 1 Data

**Fig. 1:**
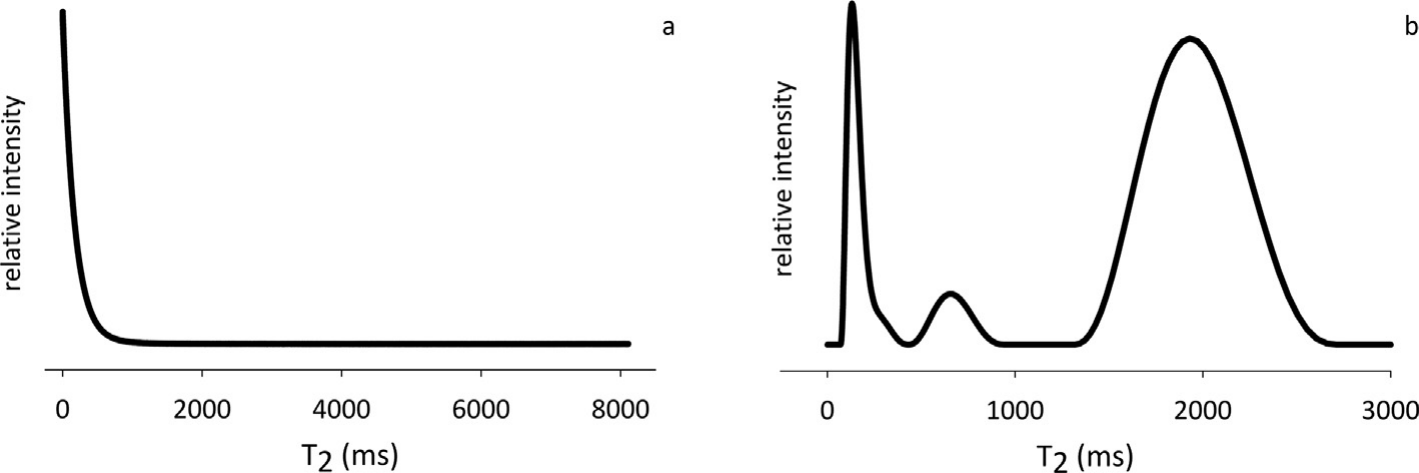
Dataset on curds during syneresis. Representative graphical examples of (a) CPMG signal decays and (b) T_2_ quasi-continuous distributions obtained by using CONTIN software.

**Fig. 2:**
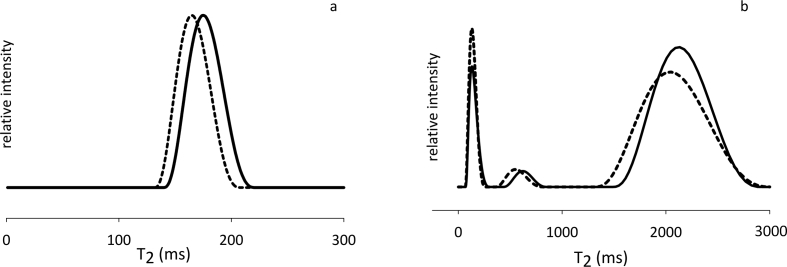
(a) T_2_ quasi-continuous distributions (CONTIN) of milk immediately after rennet addition (at T_0_) and (b) on curds after 70 min (T_6_). Solid lines refer to raw milk and derived curd, dashed lines represent the heat-treated counterparts.

TD-NMR technique showed its ability and suitability in unraveling dairy products features and quality attributes [1], [2], [3]. Heating is known to affect milk functional properties [4], [5] and, consequently, the following processing steps in cheese manufacturing. With TD-NMR being able to highlight molecular changes related to coagulation and syneresis [6], [7], a TD-NMR T_2_ dataset of whole milk is here presented, to deepen the knowledge of the syneresis process. Both raw (T_2_ decays, Fig. 1a) and processed (T_2_ distributions, Fig. 1b) data of raw and heat-treated cow's and goat's milk (after rennet addition, and up to 70 minutes of syneresis) [1] are provided as supplementary files, with an explanatory legend. Representative examples of the differences between the NMR relaxometric profiles of raw and heat-treated milk (Fig. 2a) and curd (Fig. 2b) are also reported.

## 2 Experimental design, materials, and methods

### 2.1 Sample preparation

Fresh whole raw milk was collected and heat-treated at 72 °C. Raw and heat-treated milk samples were put into NMR glass tubes and inserted inside the magnet to equilibrate at 40 °C. Liquid rennet (Naturen®, Christian Hansen, Parma, Italy) was added to milk inside the NMR tubes, where curd evolution was monitored during coagulation and syneresis.

### 2.2 Curd syneresis monitoring

T_2_ distributions were acquired at T_0_ (just after rennet addition),T_3C_ (curd cutting), and every 10 minutes until 70 minutes (T_4_, T_5_, T_6_ and T_7_).

### 2.3 TD-NMR analysis

A low-field Nuclear Magnetic Resonance spectrometer was used (Bruker the miniSpec, Germany). ^1^H T_2_ decays were acquired with a Carr-Purcell-Meiboom-Gill (CPMG) pulse sequence (recycle delay: 6 s; interpulse spacing 0.05 ms; 8 scans; 8000 data points) [Bruker the miniSpec (20MHz, 0.47T), Germany] and T_2_ quasi-continuous distributions (400 points; range: 1–3000 ms) were obtained (CONTIN Bruker software).
